# Effect of Free Ammonia, Free Nitrous Acid, and Alkalinity on the Partial Nitrification of Pretreated Pig Slurry, Using an Alternating Oxic/Anoxic SBR

**DOI:** 10.1155/2017/6571671

**Published:** 2017-09-06

**Authors:** Marisol Belmonte, Chia-Fang Hsieh, José Luis Campos, Lorna Guerrero, Ramón Méndez, Anuska Mosquera-Corral, Gladys Vidal

**Affiliations:** ^1^Engineering and Environmental Biotechnology Group, Environmental Science Faculty & Center EULA-Chile, University of Concepción, P.O. Box 160-C, Concepción, Chile; ^2^School of Biochemical Engineering, Pontificia Universidad Católica de Valparaíso, 2362803 Valparaíso, Chile; ^3^Laboratory of Biotechnology, Environment and Engineering, Faculty of Engineering, University of Playa Ancha, 2340000 Valparaíso, Chile; ^4^Facultad de Ingeniería y Ciencias, Universidad Adolfo Ibáñez, 2503500 Viña del Mar, Chile; ^5^Department of Chemical and Environmental Engineering, University Federico Santa María, 2390123 Valparaíso, Chile; ^6^Department of Chemical Engineering, School of Engineering, University of Santiago de Compostela, 15782 Santiago de Compostela, Spain

## Abstract

The effect of free ammonia (NH_3_ or FA), free nitrous acid (HNO_2_ or FNA), and total alkalinity (TA) on the performance of a partial nitrification (PN) sequencing batch reactor (SBR) treating anaerobically pretreated pig slurry was studied. The SBR was operated under alternating oxic/anoxic (O/A) conditions and was fed during anoxic phases. This strategy allowed using organic matter to partially remove nitrite (NO_2_^−^) and nitrate (NO_3_^−^) generated during oxic phases. The desired NH_4_^+^ to NO_2_^−^ ratio of 1.3 g N/g N was obtained when an Ammonium Loading Rate (ALR) of 0.09 g NH_4_^+^-N/L·d was applied. The system was operated at a solid retention time (SRT) of 15–20 d and dissolved oxygen (DO) levels higher than 3 mg O_2_/L during the whole operational period. PN mainly occurred caused by the inhibitory effect of FNA on nitrite oxidizing bacteria (NOB). Once HNO_2_ concentration was negligible, NH_4_^+^ was fully oxidized to NO_3_^−^ in spite of the presence of FA. The use of biomass acclimated to ammonium as inoculum avoided a possible effect of FA on NOB activity.

## 1. Introduction

The intensive swine production is creating scenarios where generated waste is not correctly disposed, exceeding the assimilation capability of the soil-water-plant ecosystem of the crop lands [1]. The anaerobic digestion is the most used technology to treat this kind of wastes [2]. In this process, high removal efficiencies of carbonaceous compounds contained in the wastewater are achieved while nitrogen removal is scarce, only due to biomass growth. Since the effluent from the anaerobic digester has a low C/N ratio, to perform nitrogen removal by the combination of nitrification (sequential ammonium (NH_4_^+^) oxidation to nitrite (NO_2_^−^) and nitrate (NO_3_^−^)) and denitrification (nitrate or nitrite reduction to nitrogen gas (N_2_)) processes is not economically feasible due to the requirements of organic matter. The application of the combined partial nitrification (oxidation of ammonium to nitrite with around 50% efficiency) and anammox (combination of previously generated nitrite and ammonium to produce nitrogen gas) processes could avoid this drawback. However, some studies reflected problematic situations for nitrogen removal in this way due to the presence of relevant concentrations of residual organic matter in the treated effluent [3]. In this sense, Wett et al. [4] proposed to treat a municipal wastewater, with a low C/N ratio, in a partial nitrification unit operated in alternated oxic and anoxic periods in order to promote the use of the organic matter present for denitrification. This strategy together with the control of the solid retention time (SRT) also allowed suppressing the growth of nitrite oxidizers when the unit was operated at low temperature and low ammonium concentrations and, therefore, improving the stability of partial nitrification. Moreover, as the organic matter was removed by denitrification, alkalinity was generated which partially compensated for the alkalinity consumption due to partial nitrification.

During the treatment of wastewater with high ammonium concentrations, as the effluent of pig slurry coming from the anaerobic digestion, the presence of free ammonia (NH_3_ or FA) and/or free nitrous acid (HNO_2_ or FNA) can affect the performance of the partial nitrification process. These compounds can cause inhibition of nitrifying and denitrifying bacteria and provoke the nitrite accumulation in the system [5, 6]. The nitrifying bacteria are inhibited at concentrations of FA and FNA within 0.1–150 mg NH_3_-N/L and 0.2–2.8 mg HNO_2_-N/L, respectively [5], while the effect of FNA on denitrifying bacteria was observed within 0.01–0.20 mg HNO_2_-N/L [7]. Another factor to be considered is the inlet total alkalinity/ammonium ratio (TA/NH_4_^+^-N) since it will determine the pH value inside the reactor and, therefore, the concentrations of FNA and/or FA [8–10].

In the present research the effect of FA, FNA, and total alkalinity/ammonium ratio on the performance of a partial nitrification sequencing batch reactor (SBR) operated under alternating oxic/anoxic conditions was studied. An anaerobically pretreated pig slurry and acclimated biomass to high ammonium concentrations were used as feeding and inoculum, respectively. The operational conditions were adjusted to achieve the desired nitrite to ammonium ratio in the effluent and promote the consumption of the present organic matter by means of the denitrification process.

## 2. Materials and Methods

### 2.1. Reactor SBR Description and Operational Conditions

A laboratory scale SBR with a working volume of 1.5 L and a total volume of 2.5 L was used. Dimensions of the unit were height of 540 mm (*H*), inner diameter of 77 mm (*D*), and the *H*/*D* ratio of 7. Oxygen was supplied by means of a ceramic air diffuser located at the bottom of the reactor connected to an air pump. The system was equipped with a mechanical stirrer operated at 80 RPM. The reactor was maintained in a thermostated chamber at 33 ± 2°C. The pH was not controlled and ranged between 6.2 and 8.5. A programmable logic controller (PLC) was used to control the cycle.

**Figure 1:**
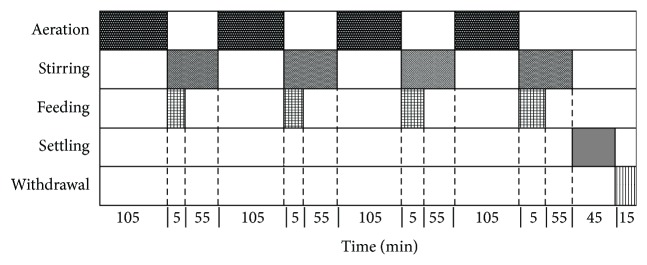
Distribution of the operational cycle.

The reactor was operated in cycles of 12 h distributed as shown in Figure 1. The volume exchange ratio was fixed at 8.3% and the hydraulic retention time (HRT) was of 6 days. The DO was supplied only during the oxic period and its concentration was kept higher than 3 mg O_2_/L. In the anoxic phase the mixture inside the reactor was achieved through mechanical stirring.

The reactor was fed with the effluent coming from an anaerobic digester treating diluted pig slurry [2], whose total alkalinity/NH_4_^+^ ratio ranged from 4.0 to 9.4 g/g. The reactor was operated during 270 days divided into three stages according to the inlet ammonium concentrations of 350, 550, and 880 mg NH_4_^+^-N/L, which corresponded to applying Ammonium Loading Rates (ALRs) of 0.06, 0.09, and 0.15 g NH_4_^+^-N/L·d, respectively (Table 1). The SRT was not controlled and ranged from 15 to 20 d during the whole operational period.

### 2.2. Activity Assays

Periodical samples of biomass were collected from the reactor during the operational stages to evaluate their specific ammonium and nitrite oxidizing activities (AOB and NOB, resp.) and specific denitrifying activity (SDA). The specific nitrifying activity (ammonium and nitrite oxidizing) of the biomass was determined by respirometric assays, applying the methodology described by López-Fiuza et al. [11], while the maximum SDA of the sludge was determined according to the methodology proposed by Buys et al. [12].

### 2.3. Inoculum

The SBR was inoculated with 5 g volatile suspended solids (VSS)/L of activated sludge collected from an aerobic reactor, used to remove both organic matter and nitrogen from pig slurry, located in the Region of the Libertador Bernardo O'Higgins, Chile. The initial specific nitrifying activities obtained for AOB and NOB were of 27 mg NH_4_^+^-N/g VSS·d and 19 mg NO_2_^−^-N/g VSS·d, respectively, and the initial SDA was of 72 mg NO_2_^−^-N/g VSS·d.

### 2.4. Analytical Methods

Concentrations of soluble chemical oxygen demand (COD_S_), VSS, NH_4_^+^, NO_2_^−^, and NO_3_^−^ were determined according to the Standard Methods [13]. Total alkalinity was determined by titration according to the methodology described by Ripley et al. [14]. The pH value and DO concentrations were measured using specific electrodes (pH: sensor Multiparameter Oakton PC650 model; DO: sensor Oxi 330 WTW).

### 2.5. Calculations

The nitrification (NIT) and nitrite accumulation ratio (NAR) percentages were calculated according to the following equations:

(1) NIT=NH4+-Ni−NH4+-NeNH4+-Ni·100

(2) NAR=NO2−-NeNO3−-Ne+NO2−-Ne·100,

where NH_4_^+^-N_*i*_ and NH_4_^+^-N_*e*_ are the concentrations of ammonia in the influent and effluent, respectively, and NO_2_^−^-N_*e*_ and NO_3_^−^-N_*e*_ are the concentrations of nitrite and nitrate in the effluent, respectively.

The ratio between the amount of organic matter removed and nitrogen removed (COD_removed_/N_removed_, g COD_S_/g N) was calculated according to the following equation:

(3) CODremovedNremoved=CODi−CODeNH4+-Ni−NO3−-Ne+NO2−-Ne+NH4+-Ne.

The concentrations of NH_3_ and HNO_2_ were calculated from the NH_4_^+^-N and NO_2_^−^-N concentrations inside the reactor, respectively, at the operating temperature and the pH value in the bulk liquid according to the expressions proposed by Hou et al. [15]. Ammonium and nitrite consumption rates were calculated from the profiles of ammonium and nitrite concentrations obtained in the reactor from the measurements performed throughout the operating cycles as described by Mosquera-Corral et al. [16].

## 3. Results and Discussion

### 3.1. Operation of the Reactor

**Figure 2:**
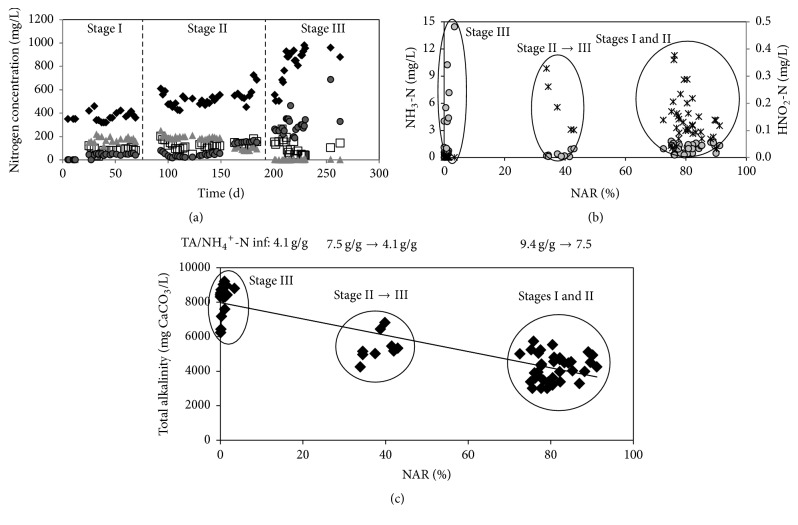
Evolution of nitrogen compounds. (a) Behavior of nitrogen concentration inside the reactor: NH_4_^+^-N influent (diamond), NH_4_^+^-N effluent (square), NO_2_^−^-N effluent (triangle), and NO_3_^−^-N effluent (circle). (b) Nitrite accumulation ratios (NAR) as percentages obtained at different HNO_2_-N (asterisk) and NH_3_-N (circle) concentrations. (c) Nitrite accumulation ratios (NAR) as percentages obtained at different total alkalinity influent (diamond) concentrations.

The partial nitrification process was developed in the reactor after 25 days of operation (Stage I). The NAR, defined as the produced nitrite to ammonium removed ratio, was of 78% (Table 1). However, the achieved NO_2_^−^-N/NH_4_^+^-N ratio in the effluent (2.1 g/g) was higher than the required value of 1.3 g/g which is considered optimal for the posttreatment of the effluent by means of the anammox process [17, 18]. The very high nitrite production, with efficiencies larger than required, indicated the system was underloaded. On day 76, ALR was increased from 0.06 to 0.09 g NH_4_^+^-N/L·d (Stage II) in order to overload the system and, therefore, to obtain the desired NO_2_^−^-N/NH_4_^+^-N ratio. In these conditions the NAR remained almost constant (83%) while the ammonium level in the effluent increased which allows obtaining a NO_2_^−^-N/NH_4_^+^-N ratio in the effluent of 1.3 g/g. On day 160 (Stage II) a failure of the aeration system caused that the system operated under anoxic conditions for more than 48 h and nitrite concentration in the reactor decreased from around 200 to 100 mg NO_2_^−^-N/L (Figure 2(a)). After this event the partial nitrification turned unstable and NAR diminished progressively while the nitrate concentration increased. In Stage III, the applied ALR was increased up to 0.15 g NH_4_^+^-N/L·d with the aim of overloading the nitrifying microorganisms and, therefore, of accumulating nitrite, but the system was able to fully oxidize ammonium to nitrate at an efficiency of 86% and the previous partial nitrification conditions were not recovered. The destabilization of the PN that occurred on day 160 could be related to the prolonged anoxic period suffered by both AOB and NOB due the failure of the aeration system. According to Torà et al. [19], the activity of AOB would decrease around 14% after two days under anoxic conditions but the decrease expected for NOB activity would be higher than that of AOB activity due to the higher value of their decay constant [20]. Then, the effect the prolonged anoxic period on the loss of PN stability could be attributed to the decrease of nitrite concentration observed during this period.

In order to evaluate the possible reasons for the loss of nitrite accumulation, the FA and FNA concentrations were estimated for the different operational stages as shown in Figure 2(b). Collected results indicate that high NAR were obtained in the presence of FNA concentrations within 0.08–0.37 mg HNO_2_-N/L. These concentrations could be responsible for the inhibition of NOB and the consequent nitrite accumulation as it has been observed by authors like Park and Bae [21] and Kim and Seo [22]. On the contrary, no correlation between nitrite accumulation and FA concentrations calculated was detected. Ammonium was fully oxidized to nitrate even at FA concentrations higher than 3 mg NH_3_-N/L, a value which is generally reported as inhibitory for NOB [5, 21, 23]. Nevertheless, if biomass is adapted to the FA, the NOB inhibition can be only achieved at FA concentrations higher than 10 mg NH_3_-N/L [24–26]. Results indicate that NAR was quickly lost once the inhibitory conditions for NOB were suppressed in Stage III. This would demonstrate that the inhibitory effects of FNA on NOB were temporary [15, 27].

Alkalinity is another important parameter that affects the nitrification process since it is important to maintain a suitable pH value [28].  Figure 2(c) shows that the decrease of the inlet total alkalinity concentrations increased the efficiency of the system in accumulating nitrite. This can be attributed to the decrease of pH value inside the reactor, which promoted the inhibitory effect of FNA on NOB (see Table 1). On the contrary, Hwang et al. [10] and Hou et al. [15] observed that nitrite was only accumulated when the inlet TA/NH_4_^+^-N ratio was higher than the stoichiometric one (7.1 g TA/g NH_4_^+^-N) since this favoured the FA.

Respirometric assays were performed with biomass samples from the reactor. Experimental results confirmed the observations from the reactor operation. The initial NOB specific activity decreased to no detectable levels on day 25 and remained at this level until day 187. These results were related to the increase in the FNA concentration and its inhibitory effect on NOB (Figure 2(b)). From this day on, NOB specific activity gradually increased up to a value of 230 mg NO_2_^−^-N/g VSS·d. The AOB specific activity was of 47, 66, and 124 mg NH_4_^+^-N/g VSS·d during Stages I, II, and III, respectively. The SDA of the biomass was of 120 mg N/g VSS·d during the whole operational period.

**Figure 3:**
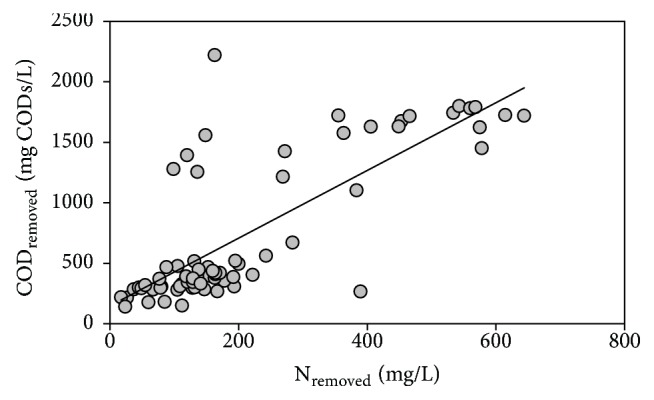
Relationship between COD_S_ and N removed throughout the operational period.

The COD removal efficiency was affected by the influent quality and ranged from 39% during Stage I to 85% during Stage III. Concerning nitrogen removal efficiency, its value ranged from 21% (Stage I) to 47% (Stage III) (Table 1). Results showed a clear relationship between the amounts of organic matter and nitrogen removed, which indicate that the denitrification process was taking place (Figure 3). During Stage II, the COD_removed_/N_removed_ ratio was of 2.6 g/g while, during Stage III, this ratio increased up to 3.5 g/g because ammonium was completely oxidized to nitrate during this stage [29].

### 3.2. Operational Cycles

**Figure 4:**
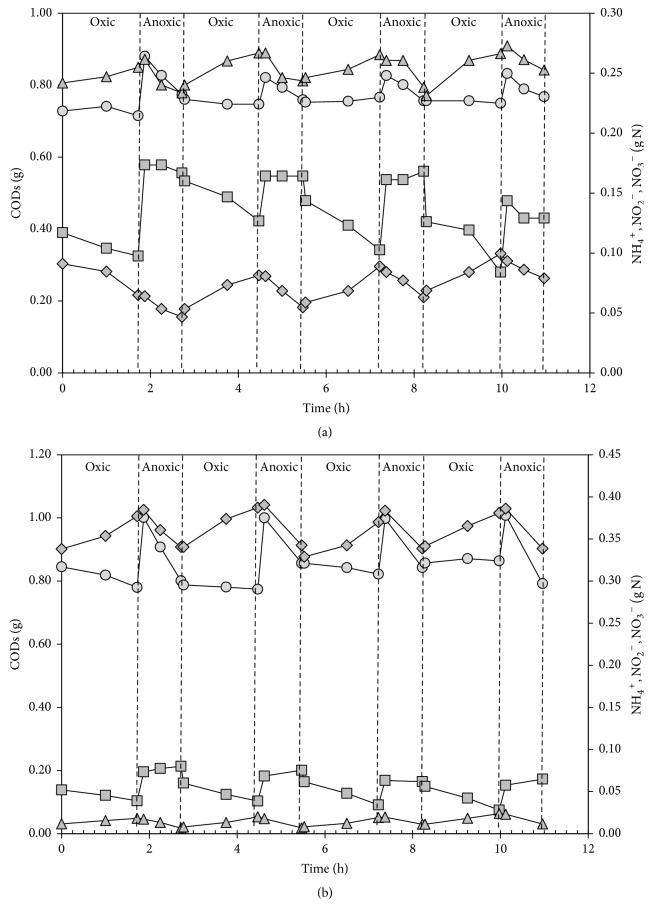
Evolution of COD_S_ (circle), NH_4_^+^-N (square), NO_2_^−^-N (triangle), and NO_3_^−^-N (diamond) calculated amounts inside the reactor during an operational cycle in the SBR on days 109 (Stage II) (a) and 221 (Stage III) (b).

In order to evaluate the performance of the reactor, cycle measurements were carried out. The COD_S_ and nitrogenous compounds concentrations were measured throughout different operational cycles over the operational period. Data obtained from the cycle measurements of days 109 (Stage II) and 221 (Stage III) are shown in Figure 4 in terms of accumulative amounts (g) of the measured compounds. In both cycles, biodegradable organic matter was mainly removed during the anoxic phases together with nitrite and nitrate while COD_S_ remained practically constant during oxic phases. These profiles show that the cycle distribution allows the efficient use of the organic matter, contained in the influent, to carry out denitrification. Therefore, the nitrogen load to be treated in a subsequent anammox reactor decreases. The main difference between both measured cycles was the relative values of ammonium and nitrite oxidation rates. On day 109, the ammonium oxidation rate was 3 to 4 times larger than the nitrite oxidation rate, while on day 229, both values were almost the same thus indicating that the partial nitrification was not possible during the last stage.

Gilbert et al. [30] observed the appearance of a lag phase longer for the nitrite oxidation than for the ammonia oxidation when anoxic periods, longer than 15–20 minutes, were imposed. However the lag phase corresponding to the nitrite oxidation had been always lower than 12 minutes. Therefore, the operational cycle distribution used in this work would not be suitable to promote PN due to the long duration of the oxic period (105 minutes). In fact, when alternating oxic/anoxic conditions are applied to avoid the nitrite oxidation during municipal wastewater treatment by means of continuous systems, where neither FA nor FNA inhibitions are expected, the length of the oxic and anoxic periods is fixed at around 10 minutes [4, 29, 31].

## 4. Conclusions

Stable partial nitrification was achieved during the treatment of the effluent of an anaerobic reactor fed with pig slurry. Since the system was operated at high STR values (15–20 d) and nonlimiting oxygen concentrations (higher than 3 mg O_2_/L), nitrite accumulation was mainly attributed to the presence of high FNA concentrations. Attempts to inhibit NOB by promoting the presence of FA were not successful.

## Figures and Tables

**Table 1:** Characterization of the different operational stages of the SBR reactor.

Parameter	Unit	Stage					
		I		II		III	
		Influent	Effluent	Influent	Effluent	Influent	Effluent
Operation time	d	0–75		76–190		191–270	
ALRs	g NH_4_^+^-N/L·d	0.06		0.09		0.15	
Total alkalinity/NH_4_^+^-N	g/g	9.4 ± 0.0	—	7.5 ± 0.0	—	4.1 ± 0.0^*∗*^	—
pH		7.5 ± 0.1	7.4 ± 1.3	7.5 ± 0.1	6.8 ± 0.9	7.5 ± 0.1	7.2 ± 1.3
COD_S_	mg/L	734 ± 85	415 ± 28	801 ± 100	363 ± 189	1907 ± 319	293 ± 58
NH_4_^+^-N	mg/L	350 ± 26	82 ± 25	550 ± 67	128 ± 77	880 ± 100	102 ± 60
NO_2_^−^-N	mg/L	<1.0	174 ± 32	<1.0	165 ± 86^*∗*^	<1.0	2 ± 2
NO_3_^−^-N	mg/L	<1.0	45 ± 5	<1.0	42 ± 17^*∗*^	<1.0	293 ± 138
NO_2_^−^-N/NH_4_^+^-N	g/g	—	2.1 ± 0.5	—	1.3 ± 0.5	—	<0.1 ± <0.1
NIT	%	79 ± 5		76 ± 5		86 ± 11	
NAR	%	78 ± 2		83 ± 6^*∗∗*^		1 ± <0.1	
N_removed_	%	21 ± 13		30 ± 7		45 ± 19	
COD_removed_	%	39 ± 9		54 ± 11		85 ± 3	
COD_removed_/N_removed_	g/g	2.8 ± 1		2.6 ± 0.4		3.5 ± 0.5	

^*∗*^Sodium bicarbonate (NaHCO_3_) was added to the influent to keep it at total alkalinity/NH_4_^+^-N ratio of 4.1 g/g. ^*∗∗*^During the stable period (days 76 to 160).

## References

[1] Fridrich B., Krčmar D., Dalmacija B. (2014). Impact of wastewater from pig farm lagoons on the quality of local groundwater. *Agricultural Water Management*.

[2] Rodriguez D. C., Belmonte M., Peñuela G., Campos J. L., Vidal G. (2011). Behaviour of molecular weight distribution for the liquid fraction of pig slurry treated by anaerobic digestion. *Environmental Technology*.

[3] Lackner S., Terada A., Smets B. F. (2008). Heterotrophic activity compromises autotrophic nitrogen removal in membrane-aerated biofilms: Results of a modeling study. *Water Research*.

[4] Wett B., Omari A., Podmirseg S. M. (2013). Going for mainstream deammonification from bench to full scale for maximized resource efficiency. *Water Science and Technology*.

[5] Anthonisen A. C., Loehr R. C., Prakasam T. B. S., Srinath E. G. (1976). Inhibition of nitrification by ammonia and nitrous acid. *Research Journal Water Pollution Control*.

[6] Almeida J. S., Júlio S. M., Reis M. A. M., Carrondo M. J. T. (1995). Nitrite inhibition of denitrification by Pseudomonas fluorescens. *Biotechnology and Bioengineering*.

[7] Zhou Y., Oehmen A., Lim M., Vadivelu V., Ng W. J. (2011). The role of nitrite and free nitrous acid (FNA) in wastewater treatment plants. *Water Research*.

[8] Durán U., Val Del Río A., Campos J. L., Mosquera-Corral A., Méndez R. (2014). Enhanced ammonia removal at room temperature by pH controlled partial nitrification and subsequent anaerobic ammonium oxidation. *Environmental Technology (United Kingdom)*.

[9] Zhang L., Yang J., Hira D., Fujii T., Furukawa K. (2011). High-rate partial nitrification treatment of reject water as a pretreatment for anaerobic ammonium oxidation (anammox). *Bioresource Technology*.

[10] Hwang B.-H., Hwang K.-Y., Choi E.-S., Choi D.-K., Jung J.-Y. (2000). Enhanced nitrite build-up in proportion to increasing alklinity/NH_4_^+^ ratio of influent in biofilm reactor. *Biotechnology Letters*.

[11] López-Fiuza J., Buys B., Mosquera-Corral A., Omil F., Méndez R. (2002). Toxic effects exerted on methanogenic, nitrifying and denitrifying bacteria by chemicals used in a milk analysis laboratory. *Enzyme and Microbial Technology*.

[12] Buys B. R., Mosquera-Corral A., Sánchez M., Méndez R. (2000). Development and application of a denitrification test based on gas production. *Water Science and Technology*.

[13] APHA-AWWA-WPCF (2005). *Standard Methods for Examination of Water and Wastewater*.

[14] Ripley L. E., Boyle W. C., Converse J. C. (1986). Improved alkalimetric monitoring for anaerobic digestion of high-strength wastes. *Journal of the Water Pollution Control Federation*.

[15] Hou B., Han H., Jia S., Zhuang H., Zhao Q., Xu P. (2014). Effect of alkalinity on nitrite accumulation in treatment of coal chemical industry wastewater using moving bed biofilm reactor. *Journal of Environmental Sciences (China)*.

[16] Mosquera-Corral A., De Kreuk M. K., Heijnen J. J., Van Loosdrecht M. C. M. (2005). Effects of oxygen concentration on N-removal in an aerobic granular sludge reactor. *Water Research*.

[17] Ahn Y.-H. (2006). Sustainable nitrogen elimination biotechnologies: a review. *Process Biochemistry*.

[18] Vázquez-Padín J., Fernádez I., Figueroa M., Mosquera-Corral A., Campos J.-L., Méndez R. (2009). Applications of Anammox based processes to treat anaerobic digester supernatant at room temperature. *Bioresource Technology*.

[19] Torà J. A., Lafuente J., Baeza J. A., Carrera J. (2011). Long-term starvation and subsequent reactivation of a high-rate partial nitrification activated sludge pilot plant. *Bioresource Technology*.

[20] Munz G., Lubello C., Oleszkiewicz J. A. (2011). Factors affecting the growth rates of ammonium and nitrite oxidizing bacteria. *Chemosphere*.

[21] Park S., Bae W. (2009). Modeling kinetics of ammonium oxidation and nitrite oxidation under simultaneous inhibition by free ammonia and free nitrous acid. *Process Biochemistry*.

[22] Kim D.-J., Seo D. (2006). Selective enrichment and granulation of ammonia oxidizers in a sequencing batch airlift reactor. *Process Biochemistry*.

[23] Li J., Elliott D., Nielsen M., Healy M. G., Zhan X. (2011). Long-term partial nitrification in an intermittently aerated sequencing batch reactor (SBR) treating ammonium-rich wastewater under controlled oxygen-limited conditions. *Biochemical Engineering Journal*.

[24] Spagni A., Marsili-Libelli S. (2009). Nitrogen removal via nitrite in a sequencing batch reactor treating sanitary landfill leachate. *Bioresource Technology*.

[25] Belmonte M., Hsieh C.-F., Figueroa C., Campos J. L., Vidal G. (2011). Effect of free ammonia nitrogen on the methanogenic activity of swine wastewater. *Electronic Journal of Biotechnology*.

[26] Shanahan J. W., Semmens M. J. (2015). Alkalinity and pH effects on nitrification in a membrane aerated bioreactor: an experimental and model analysis. *Water Research*.

[27] Chai L.-Y., Ali M., Min X.-B. (2015). Partial nitrification in an air-lift reactor with long-term feeding of increasing ammonium concentrations. *Bioresource Technology*.

[28] Pijuan M., Ye L., Yuan Z. (2010). Free nitrous acid inhibition on the aerobic metabolism of poly-phosphate accumulating organisms. *Water Research*.

[29] Ge S., Peng Y., Qiu S., Zhu A., Ren N. (2014). Complete nitrogen removal from municipal wastewater via partial nitrification by appropriately alternating anoxic/aerobic conditions in a continuous plug-flow step feed process. *Water Research*.

[30] Gilbert E. M., Agrawal S., Brunner F., Schwartz T., Horn H., Lackner S. (2014). Response of different Nitrospira Species to anoxic periods depends on operational DO. *Environmental Science and Technology*.

[31] Regmi P., Miller M. W., Holgate B. (2014). Control of aeration, aerobic SRT and COD input for mainstream nitritation/denitritation. *Water Research*.

